# Estimation of Variance Components and Genomic Prediction for Individual Birth Weight Using Three Different Genome-Wide SNP Platforms in Yorkshire Pigs

**DOI:** 10.3390/ani10122219

**Published:** 2020-11-26

**Authors:** Jungjae Lee, Sang-Min Lee, Byeonghwi Lim, Jun Park, Kwang-Lim Song, Jung-Hwan Jeon, Chong-Sam Na, Jun-Mo Kim

**Affiliations:** 1 Department of Animal Science and Technology, College of Biotechnology and Natural Resources, Chung-Ang University, Anseong, Gyeonggi-do 17546, Korea; jungjae.ansc@gmail.com (J.L.); hwi1208@cau.ac.kr (B.L.); 2 Jung P&C Institute, Inc., 1504 U-TOWER, Yongin-si, Gyeonggi-do 16950, Korea; 3 Department of Animal Biotechnology, Jeonbuk National University, Jeonju, Jeollabuk-do 54896, Korea; iq6000@naver.com (S.-M.L.); jpbreed90@naver.com (J.P.); 4 Kaya Breeding Station, Sacheon, Gyeongsangnam-do 52510, Korea; barona93@daum.net; 5 Animal Welfare Research Team, National Institute of Animal Science, RDA, Wanju, Jeollabuk-do 55365, Korea; jeon75@korea.kr

**Keywords:** individual birth weight, single nucleotide polymorphism, genome-wide association studies, genomic prediction, Yorkshire pigs

## Abstract

**Simple Summary:**

The individual birth weight (IBW) of pigs is an important trait regarding its relevance to mortality at weaning, sow prolificacy, and growth performance. This study investigates the variance component estimation, informative window regions, and the efficiency of genomic predictions associated with IBW traits in Yorkshire pigs. The low heritability (0.13) is estimated on the basis of a full model including individual genetic, sow genetic, and common environmental effects. Two common window regions of the genome are identified under three different genotyping platforms found within the *ARAP2* and *TSN* genes concerning the IBW trait. The genomic prediction ability is improved using deregressed estimated breeding values including parental information as a response variable despite Bayesian methods and genotyping platforms for the IBW trait in Korean Yorkshire pigs.

**Abstract:**

This study estimates the individual birth weight (IBW) trait heritability and investigates the genomic prediction efficiency using three types of high-density single nucleotide polymorphism (SNP) genotyping panels in Korean Yorkshire pigs. We use 38,864 IBW phenotypic records to identify a suitable model for statistical genetics, where 698 genotypes match our phenotypic records. During our genomic analysis, the deregressed estimated breeding values (DEBVs) and their reliabilities are used as derived response variables from the estimated breeding values (EBVs). Bayesian methods identify the informative regions and perform the genomic prediction using the IBW trait, in which two common significant window regions (SSC8 27 Mb and SSC15 29 Mb) are identified using the three genotyping platforms. Higher prediction ability is observed using the DEBV-including parent average as a response variable, regardless of the SNP genotyping panels and the Bayesian methods, relative to the DEBV-excluding parent average. Hence, we suggest that fine-mapping studies targeting the identified informative regions in this study are necessary to find the causal mutations to improve the IBW trait’s prediction ability. Furthermore, studying the IBW trait using a genomic prediction model with a larger genomic dataset may improve the genomic prediction accuracy in Korean Yorkshire pigs.

## 1. Introduction

Litter size and birth weight have been used as representative traits of sow productivity [1]. However, pigs have been selectively bred to focus only on litter size to improve sow productivity [2]. According to the 2019 Korean pig industry report of the Korea Animal Improvement Association (KAIA), the total number of living piglets per litter has increased from 10.5 to 12.1 in the past 10 years, indicating that sow prolificacy has improved. However, it has led to an increase in the occurrence of intra-uterine growth restriction due to the lack of available capacity, oxygen, and nutrients in the uterus and, in turn, causes the birth weight variation to increase [3,4]. Owing to this association, weaning mortality is a major issue for continuously improving sow prolificacy, as it is known to affect the early growth performance. One of the most representative traits is individual birth weight (IBW) in piglets [5,6,7,8], where IBW reflects the individual abilities of the offspring that are affected by genetic factors and the intrauterine nutritional supply. Hence, improving the genetic progress of IBW is crucial for understanding the genetic control of the indicators [9]. However, previous studies have reported that the heritability of the birth weight is low, ranging from 0.09 to 0.21 [10,11,12]. Therefore, it is a worthwhile endeavor to identify which genes underlie this heritability effect on birth weight. Several studies have reported numerous genetic effects on birth weight [5], where they analyzed 14,226 Yorkshire and 12,313 Landrace sows and identified candidate genes using genome-wide association studies (GWAS) such as SKOR2, SMAD2, VAV3, and NTNG1. Moreover, candidate genes such as *MYOG*, *MSTN*, and *DBH* have been reported to affect birth weight [13,14,15]. Genomic selection (GS) methodology also has been widely applied in livestock species, including dairy cattle [16], poultry [17], beef cattle [18], sheep [19], and pigs [20], using genomic information obtained from commercial single nucleotide polymorphism (SNP) genotyping platforms such as Illumina (https://www.illumina.com/products/by-type/microarray-kits/porcine-snp60.html), Neogen GeneSeek (https://genomics.neogen.com/en/ggp-porcine), and Affymetrix (https://genomics.neogen.com/en/affymetrix-axiom-porcine-array). Regarding the birth weight trait in pigs, a previous study [21] identified 17 genomic regions associated with birth weight, whereas another study [22] found 12 SNPs that were significantly associated with piglet uniformity, in which 27 differentially selected regions that were associated with the birth weight of piglets were detected by another study [23]. However, to the best of our knowledge, this is the first study to focus on assessing IBW for estimating variance components by observing the direct and maternal genetic effects as well as applying genomic analysis in the Korean pig industry. Therefore, the objective of this study is to focus on the Korean Yorkshire pigs to (1) estimate their IBW trait variance components and heritability, (2) identify the informative regions through GWAS, and (3) compare the genomic prediction abilities of two methods under two response variables using three SNP genotyping platforms.

## 2. Materials and Methods

### 2.1. Descriptive Statistics of the Phenotype and Pedigree

Here, the IBW trait was recorded between 2005 and 2020 in the GGP farm in Korea. The pedigree and phenotypic records of 76,108 and 38,864 Yorkshire pigs, respectively, were used to estimate the variance components and the genetic parameters to identify the most optimal statistical model. The average and standard deviation of IBW were 1.372 ± 0.284 kg, ranging from 0.60 kg to 2.70 kg.

### 2.2. Genotypic Data Editing and Imputation

A total of 3858 Korean Yorkshire pig genotypes were used as a reference set across three SNP genotyping platforms of Illumina porcineSNP60 v2 (Illumina, Inc., San Diego, CA, USA) and Axiom porcine55K and Axiom porcine660K (Affymetrix, Inc., Santa Clara, CA, USA), which consisted of 61,565, 55,372, and 658,692 SNP markers, respectively. Quality control measures of the SNP markers and animals included the exclusion of unmapped SNPs, sex chromosome SNPs, SNPs with a poor call rate (<0.90), and duplicate SNP map-positions. Additionally, animals with a poor call rate (<0.90) were excluded, as well as animals that did not have a registration number and animals with a poor call rate for duplicate genotypes within and among genotyping platforms. Consequently, the number of available SNP markers was 47,567, 52,398, and 539,459 across the 55K, 60K, and 660K SNP panels, respectively, using the Sscrofa 11.1 mapping information. This resulted in 429, 2,579, and 406 animals across the 55K, 60K, and 660K SNP panels, respectively, which were used in the GWAS and genomic prediction modeling. Briefly, the imputation process of the three SNP genotyping platforms was performed separately as follows: all the genotypes were imputed to their respective SNP genotyping panels using the FImpute v2.2 software [24] with default settings. Then, we accepted the three different non-missing genotypes in the imputed 55K, 60K, and 660K data that consisted of 3414 animals.

### 2.3. Estimating the Variance Components and the Genetic Parameters

#### 2.3.1. Model Definition

To establish an optimal statistical model for estimating the variance components and the genetic parameters of the Yorkshire pigs’ IBW, four animal models that considered the maternal genetic effects, the common environmental effects, and the animal genetic effects, in addition to the fixed and covariance effects were used. The birth weight of each individual was used as a response variable, in which the first model (Equation (1)) applied an animal model that only considered the individual genetic effect, whereas the second model (Equation (2)) considered the maternal genetic effect, and the third model (Equation (3)) considered the common environmental effect that occurs during the pregnancy of full-siblings. Finally, the fourth model (Equation (4)) applied a full model that included the sow genetic effect and the common environment effect as well as the individual genetic effect. During this statistical model, the effects of sex, parity of dam, and contemporary group (farm-year-season) were included as fixed effects. To correct the IBW, which is a response variable, the litter size of dams was applied to each model as a covariate term effect, in which the variance components and the genetic parameters were estimated by fitting all the models using the restricted maximum likelihood (REML) procedure that was implemented in the statistical software R using the ASReml v4.1 package [25]. The four statistical models were as follows:

Equation (1): Animal model with a direct additive genetic effect:

(1) $$y=\mathrm{Xb}+Z_{a}u_{a}+e$$

Equation (2): Animal model with a direct additive genetic effect and a maternal genetic effect:

(2) $$y=\mathrm{Xb}+Z_{a}u_{a}+Z_{m}u_{m}+e$$

Equation (3): Animal model with a direct additive genetic effect and a common litter effect:

(3) $$y=\mathrm{Xb}+Z_{a}u_{a}+W_{C}+e$$

Equation (4): Animal model with a direct additive genetic effect, a maternal genetic effect, and a common litter effect:

(4) $$y=\mathrm{Xb}+Z_{a}u_{a}+Z_{m}u_{m}+W_{c}+e$$

where, y is the vector of observations (IBW); $X,Z_{a},Z_{d},W$ are known incidence matrices that relate the observations to fixed and random effects; b is the vector of fixed effects; $u_{a}$ is the vector of direct additive genetic effects; $u_{m}$ is the vector of maternal genetic effects; c is the vector of common non-genetic effects; and e is the vector of the residuals.

#### 2.3.2. The Likelihood Ratio Test

To determine the most suitable model for estimating the genetic parameters, the log-likelihood values (LogL) of the four different models were compared.

The Chi-Square statistical test ($x^{2}$) was used accordingly [26] and was calculated as follows:

$$x^{2}=-2\left(LogL_{reducedmodel}-LogL_{fullmodel}\right)$$

where, $LogL_{reducedmodel}$ is the log-likelihood of a simpler model, and $LogL_{fullmodel}$ is the log-likelihood of a more complete model.

### 2.4. Deregressed Estimated Breeding Values of the Response Variables in Genomic Analysis

The genetic parameters, the estimated breeding values (EBVs), and the reliability of each individual were estimated by a single-trait animal model accompanied by a full model (Equation (4)) using the ASReml v4.1 package in R [25]. Two types of deregressed estimated breeding values (DEBVs), which were (1) a combination of deregression and adjustment for ancestral information (DEBVexcPA), and (2) the parent average EBV (PA) added back to the DEBVs (DEBVincPA) [27], were estimated based on these EBVs and their reliabilities in terms of their response variables in the genomic analysis. A detailed description of these DEBVs was provided [20]. Moreover, the weighting factor ($w_{i}$) of each individual in the genomic analysis also was calculated by applying a previously proposed model [27]. Finally, the individuals with a reliability of less than 0.01 were removed, and the 678 registered Yorkshire pigs with their genomic and phenotypic data were used in the genomic analysis.

### 2.5. Statistical Method for Estimating SNP Effects

Two Bayesian methods, BayesB [28] and BayesC [29], used different π values of 0.99 when using medium-density genotyping platforms and 0.999 when using a high-density genotyping platform. The weighting factors were used to estimate the SNP marker effects using the GenSel4R software [30] for genomic analysis in the GWAS and genomic prediction. The two Bayesian methods were fitted using the following equation:

$$y_{i}=\mu +\overset{k}{\underset{j=1}{\sum }}Z_{ij}u_{j}\delta_{j}+e_{i}$$

where, $y_{i}$ is the response variable (DEBVs); μ is the population mean; k is the number of markers; $Z_{ij}$ is the allelic state (−10, 0, and 10 in GenSel4R) of the $j^{th}$ marker of the $i^{th}$ individual; $u_{j}$ is the SNP maker effect at locus j in an individual; and $\delta_{j}$ indicates the presence or absence (0 or 1) of the SNP marker in the model. To estimate the effect and the SNP marker variance, which were estimated by the posterior mean and included the posterior distributions of the parameters and the effects obtained using the Gibbs sampling, the initial 10,000 iterations out of a total of 110,000 Markov chain Monte Carlo (MCMC) iterations were excluded as a part of the burn-in period with a thinning interval of five iterations. All the genomic analyses were performed using the GenSel4R software [30].

### 2.6. Genomic Prediction Accuracy under Five-Fold Cross-Validation

During this study, a five-fold cross-validation method was used to estimate the genome accuracy, and the k-means clustering method was used for cross-validation. The reference set with both phenotypes and genotypes was divided into the training and validation sets, in which the relatedness was configured to be as small as possible through the k-means clustering method [31]. The clustering results using 1959 elements of the pedigree information related to the genotyped animals are presented in Table 1, which was successfully partitioned, whereby the values of $a_{max}$ and $a_{ij}$ within the groups were relatively higher than the values of $a_{max}$ and $a_{ij}$ between the groups. Additionally, the genomic accuracy was calculated using the simple correlations between the direct genomic values (DGVs) via the summation of the SNP effects of the genotyped animals in each validation set and their response variables $r\left(\hat{y},y\right)$, where $\hat{y}$ is a vector of DGVs of the corresponding animals in *y*, and *y* is a vector of one of the response variables (DEBVexcPA or DEBVincPA) of the validation set.

## 3. Results and Discussion

### 3.1. Heritability and the Optimized Model

Using the four models, the optimal model for IBW was found through the likelihood ratio test, in which the estimated variance component and genetic parameter result of each model, as well as the comparison result for identifying the most optimal model, are summarized in Table 2. As shown, the heritability estimates of the IBW trait with their associated standard errors were found to be 0.500 (0.013), 0.212 (0.018), 0.322 (0.017), and 0.130 (0.019) per statistical model (Equations (1)–(4)). These results are similar to a recent report [9] when using the full model (Equation (4)) with an estimated IBW heritability in the Yorkshire and Landrace pigs of 0.15 and 0.05, respectively, while including the maternal, common litter, and direct additive effects. Regarding the case of birth weight, it was estimated that the maternal effect, which included the common environmental effect, had a fairly high variance component and, as a result of the likelihood ratio test, the full model (Equation (4)) included all the maternal effects and, hence, was identified to be the most optimal model. Moreover, the analysis result was the same as the simulation result, in which the most accurate variance components and genetic parameters can be estimated when the model considers not only the direct additive genetic effects but, also, the maternal genetic and environmental effects [32]. Through the results of this study, the most optimal statistical model for estimating the birth weight of Yorkshire pigs was identified, after which the genetically superior individuals in terms of their birth weights could be selected through the genetic parameters and breeding values that were estimated by the optimal model.

### 3.2. GWAS of the Individual Birth Weight Trait

**Figure 1:**
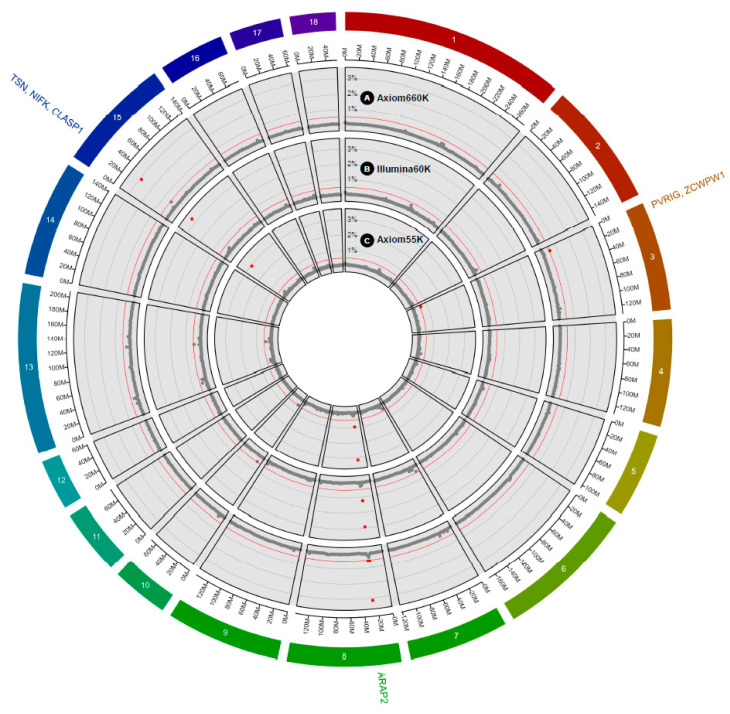
A circular Manhattan plot of the window variances of the individual birth weight trait using the three genotyping platforms, where the (A) outer, (B) middle, and (C) inner radii reflect Axiom650K, Illumina60K, and Axiom55K, respectively. The red horizontal lines and the red dots represent 0.5% of the window variance and the windows above the 0.5% threshold, respectively. Genes corresponding to the common significant windows across the genotyping platforms have been indicated outside of the plot. The gene annotations include ArfGAP with RhoGAP domain ankyrin repeat and PH domain 2 (ARAP2), Translin (TSN), nucleolar protein interacting with the FHA domain of MKI67 (NIFK), PVR-related immunoglobulin domain containing (PVRIG), zinc finger CW-type and PWWP domain containing 1 (ZCWPW1), and the cytoplasmic linker-associated protein 1 (CLASP1).

To identify the most informative 1 Mb window regions (over 0.5% of the additive genetic variance) as well as the significant SNP markers, we based our estimations on the model frequency within these window regions that were used in the GWAS analysis of the IBW trait across three commercially developed porcine SNP genotyping platforms (Illumina porcine60v2, Axiom porcine55K, and Axiom porcine660K). We employed the BayesB method with a large value of π (medium-density: 0.99 and high-density: 0.999) with DEBVincPA acting as a response variable in the Yorkshire pigs. The results of these association studies of the IBW trait are summarized in Table 3, which include the informative regions and the SNP markers that are presented in Figure 1 in the form of circular Manhattan plots across the three genotyping platforms. Considering the Axiom55K and Illumina60K panels, four informative windows were detected, respectively, whereas in Axiom660K panel, two windows were detected.

We identified 10 significant SNPs in the four windows detected by the Axiom55K, which were the same number of markers in the Illumina60K, whereas in the Axiom660K, we identified nine significant SNPs in the two detected windows. To summarize, the most significant detected window in each panel was on chromosome SSC8 at 27 Mb using the Axiom55K, on chromosome SSC15 at 29 Mb using the Illumina60K, and on chromosome SSC8 at 27 Mb using the Axiom660K platform, which explained 3.17%, 3.31%, and 3.16% of the variance, respectively. Concerning the Axiom55K panel, the most significant SNP marker (AX-116342380), which was based on the model frequency of the SNP markers, was located on SSC8 at 27.89 Mb within the *ARAP2* gene. These results were similar when using the Axiom660K genotyping platform, in which the most significant SNP marker was AX-116342374, which was located within the *ARAP2* gene. Moreover, when using the Illumina60K genotyping platform, we found that the DBWU0000855 SNP marker located on SSC15 (29.71 MB) was the most significant, which was located within the *TSN* gene. However, we also found the same informative region (SSC8 at 27 Mb) using the two Axiom genotyping platforms with the Illumina60K genotyping platform. Hence, across the three genotyping platforms, the highest percentage of additive genetic variance (3.31%) was found in Illumina 60K, whereas the Axiom55K (3.17%) and Axiom660K (3.16%) had similar values. Taken together, we identified consistent informative regions (SSC8 at 26 or 27 Mb and SSC15 at 29 Mb) with same gene annotations (*ARAP2* and *TSN*) in all platforms. Fine-mapping is needed to identify causal variants for IBW in SSC8 at 26–27 Mb in the future. The *ARAP2* (ArfGAP with RhoGAP domain, ankyrin repeat and PH domain 2) gene is known to affect the actin cytoskeleton through mediating RhoA [33]. Especially, β-actin has been reported as essential for embryogenesis and has a critical role during cell migration in a mice knockout model [34]. Additionally, the *TSN* (translin) gene is known to act as a modulator of mesenchymal cell proliferation and differentiation in mice [35]. Taken together, we suggest that significant markers located in the ARAP2 and TSN genes affect embryogenesis and cell proliferation in the fetus, resulting in the difference in porcine IBW.

### 3.3. Accuracy of the Direct Genomic Values

The genomic prediction accuracy of the studied IBW trait across the three genotyping platforms, two response variables, and two Bayesian methods are summarized in Table 4. Applying BayesB to estimate the genomic prediction accuracy using Axiom55K, Illumina60K v2, and Axiom660K resulted in 0.188, 0.168, and 0.163 and 0.261, 0.224, and 0.223 for DEBV-excluding parent average (DEBVexcPA) and DEBV-including parent average (DEBVincPA), respectively. A similar trend also was observed when applying BayesC using Axiom55K, Illumina60K v2, and Axiom660K, which resulted in 0.178, 0.157, and 0.150 and 0.252, 0.210, and 0.215 for DEBVexcPA and DEBVincPA, respectively. These results indicated there was a 1% difference in the genomic prediction accuracy between BayesB and BayesC, in which the difference among the genotyping platforms was the highest in Axiom55K, followed by Illumina60K v2 and Axiom660K. Additionally, the difference between the DEBVexcPA and DEBVincPA response variables was estimated to be 6.3%, which was higher on average when using DEBVincPA across all genotyping platforms and Bayesian methods.

Contrasting to our expectation, a high extent of linkage disequilibrium (LD) between SNP and quantitative trait loci (QTL) via increasing marker density having the potential to improve prediction accuracy [36,37] was not realized using a high-density genotyping platform such as Axiom porcine660K. However, similar trends also were reported when comparing the prediction accuracy by the density of the genotyping platforms [38,39,40,41]. Additionally, prediction ability on genotyping platforms relies on the extent of causal variants such as QTL, when targeting economical traits, which is in contrast to using dense genotyping platforms [36,42] such as 80K, 650K, and SEQ. However, with a higher proportion of genetic variance using the Axiom porcine660K genotyping platform compared with other medium-density platforms, the novel informative windows were not observed considering our GWAS results, which means that the high-density platform had no additional informative SNPs associated with the IBW trait compared with the medium-density platforms. Another potential reason for the lack of improvements in the prediction accuracy when using a high-density genotyping platform is that it could be affected by the ratio between the number of markers and the sample size [43]. Therefore, a larger sample size may be more effective when using high-density genotyping platforms.

During the current study, we observed an increase in the genomic accuracy when using DEBVincPA as a response variable as compared to DEBVexcPA across the SNP genotyping platforms and Bayesian methods. These results are consistent with previous reports in terms of growth and productive traits in Duroc pigs [40]. During our genomic prediction model, when DEBVexcPA was used as a response variable, it had the advantage of avoiding double counting issues, but the genomic accuracy was higher when DEBVincPA was used as the response variable. This can be explained by accounting for the differences in PA among the genotyped animals when including PA after deregressing from the EBVs [44]. Moreover, the obtained genomic prediction accuracy of the IBW trait was relatively small compared to previous studies conducted on the exterior growth and the productive traits of Korean pig populations [20,40]. However, these results were from a relatively small reference size; thus, continuous genetic evaluations using a larger reference size would increase the genomic accuracy of the IBW trait.

## 4. Conclusions

During this study, we estimated a low heritability and identified candidate genes of the IBW trait of Korean Yorkshire pigs; hence, applying genomic technology can rapidly improve this trait. To realize this improvement, we evaluated and compared the genomic prediction ability across various genotyping platforms, response variables, and Bayesian methods. Altogether, two common informative 1 Mb window regions were identified across the three genotyping platforms, where the prediction ability was higher when using DEBVincPA as a response variable relative to using the other response variable. Thus, we suggest that future fine-mapping studies should include target sequencing of informative regions to identify the causal mutations and improve the prediction ability of IBW traits. Furthermore, a genomic prediction model that encompasses a larger genomic dataset reflecting the IBW trait may be useful in future genetic evaluations of Korean Yorkshire pigs.

## Figures and Tables

**Table 1:** Relationship comparisons among Korean Yorkshire pigs within and across clusters.

Clusters	No. of Animals	inBreC ^1^	*a*_max_within_^2^	*a*_max_between_^3^	*a*_ij_within_^4^	*a*_ij_between_^5^
1	99	0.028	0.524	0.393	0.243	0.061
2	133	0.015	0.497	0.344	0.177	0.047
3	192	0.008	0.446	0.348	0.043	0.032
4	145	0.014	0.441	0.409	0.089	0.050
5	109	0.001	0.476	0.402	0.132	0.031
Avg.		0.013	0.477	0.379	0.137	0.044

^1^ inBreC = the average of inbreeding coefficients within each cluster. ^2^ *a*_max_within_ = the average of *a*_max_ (the maximum of relationships [*aij*] for each animal) values within each cluster. ^3^ *a*_max_between_ = the average of *a*_max_ values between the clustered groups. ^4^ *a*_ij_within_ = the average of *a*_ij_ (relationships) values within each cluster. ^5^ *a*_ij_between_ = the average of *a*_ij_ values between clustered groups.

**Table 2:** Estimates of the variance components, the direct heritability (*h*^2^), the standard error (SE), the log-likelihood values (LogL), and the likelihood ratio test (LRT) values when comparing the four models of the individual birth weight trait in Korean Yorkshire pigs.

Model ^1^	Variance Components ^2^					*h*^2^ ± SE	LogL ^3^	LRT ^4^	Model Comparison^5^	P ^6^
	$\sigma_{a}^{2}$	$\sigma_{m}^{2}$	$W_{c}$	$\sigma_{e}^{2}$	$\sigma_{p}^{2}$					
Equation (1)	0.036	-	-	0.036	0.072	0.50 ± 0.013	2156.38	3678.84	1 vs. 4	<0.0001
Equation (2)	0.015	0.018	-	0.039	0.071	0.21 ± 0.018	3140.04	2695.18	2 vs. 4	<0.0001
Equation (3)	0.022	-	0.020	0.027	0.069	0.32 ± 0.017	5581.12	254.1	3 vs. 4	<0.0001
Equation (4)	0.009	0.010	0.017	0.030	0.065	0.13 ± 0.019	5835.22			

^1^ Equation (1). Direct additive genetic effect; Equation (2). Direct additive genetic effect and maternal genetic effect; Equation (3). Direct additive genetic effect and common litter effect; Equation (4). Direct additive genetic effect, maternal genetic effect, and common litter effect (full model). ^2^ $\sigma_{a}^{2}$ = direct additive genetic variance; $\sigma_{m}^{2}$ = maternal genetic variance; $W_{c}$ = common litter variance; $\sigma_{e}^{2}$ = residual variance; $\sigma_{p}^{2}$ = phenotype variance. ^3^ LogL = Log likelihood value. ^4^ LRT = $x^{2}$ test statistic for the likelihood ratio test. ^5^ Model comparison = Equations (1)–(3) vs. Equation (4). ^6^ P = *p*-value.

**Table 3:** Summary of GWAS in each genotyping platform with the significant 1 Mb windows that were associated with the individual birth weight (IBW) trait in Korean Yorkshire pigs.

Genotyping Platform	SSC_Mb	GV (%) ^1^	Informative SNP	Position (Mb)	Genetic Effect	Model Frequency	Region Annotation	Gene Annotation ^2^
Axiom Porcine 55K	8_27	3.17	AX-116342380	27.89	−0.004	0.104	Intronic	ARAP2
			AX-116690854	27.84	0.002	0.056	Intronic	ARAP2
			AX-116342258	27.44	−0.002	0.050	Intergenic	ARAP2(dist = 333210)
			AX-116342267	27.48	−0.002	0.050	Intergenic	ARAP2(dist = 333210)
			AX-116342268	27.48	−0.001	0.049	Intergenic	ARAP2(dist = 333210)
	15_29	2.95	AX-116536263	29.71	0.005	0.118	Intronic	TSN
			AX-116674286	29.64	0.004	0.093	Intergenic	TSN(dist = 37022)
			AX-116536281	29.78	−0.003	0.074	Intronic	NIFK
	8_26	0.96	AX-116690835	26.54	0.002	0.067	Intergenic	ARAP2(dist = 1271371)
	3_8	0.57	AX-116718059	8.28	0.003	0.061	Intergenic	PVRIG(dist = 49034), ZCWPW1(dist = 41321)
Illumina 60K	15_29	3.31	DBWU0000855	29.71	0.006	0.1299	Intronic	TSN
			H3GA0044096	29.64	0.004	0.0934	Intergenic	TSN(dist = 37022)
			ALGA0084705	29.73	−0.002	0.0649	Intronic	NIFK
			ALGA0084700	29.82	-0.001	0.0525	Intronic	CLASP1
	8_27	2.91	ALGA0047127	27.89	−0.005	0.1284	Intronic	ARAP2
			ALGA0047120	27.86	0.002	0.0726	Intronic	ARAP2
			ALGA0047098	27.48	−0.002	0.0636	Intergenic	ARAP2(dist = 294518)
			ALGA0047102	27.52	0.001	0.0557	Intergenic	ARAP2(dist = 256059)
	8_26	1.24	INRA0029430	26.50	0.002	0.0783	Intergenic	ARAP2(dist = 1271371)
	7_9	0.41	ALGA0107233	9.29	0.001	0.0493	Intronic	PHACTR1
Axiom porcine 660K	8_27	3.16	AX-116342374	27.87	0.001	0.021	Intronic	ARAP2
			AX-116342286	27.54	−0.001	0.016	Intergenic	ARAP2(dist = 229771)
			AX-116342230	27.31	0.001	0.012	Intergenic	ARAP2(dist = 456854)
			AX-116342377	27.88	0.001	0.012	Intronic	ARAP2
	15_29	2.89	AX-116762423	29.65	−0.001	0.023	Intergenic	TSN(dist = 22517)
			AX-116744904	29.65	0.001	0.022	Intergenic	TSN(dist=22517)
			AX-116536258	29.71	−0.001	0.014	Intronic	NIFK
			AX-116536264	29.72	−0.001	0.012	Intronic	NIFK
			AX-116536266	29.73	0.001	0.012	Intronic	NIFK

^1^ GV(%) = Percentage of additive genetic variance explained by SNP markers within each 1 Mb window region; ^2^ ARAP2 = The gene annotations include ArfGAP with RhoGAP domain ankyrin repeat and PH domain 2, TSN = Translin, NIFK = nucleolar protein interacting with the FHA domain of MKI67, PVRIG = PVR related immunoglobulin domain containing, ZCWPW1 = zinc finger CW-type and PWWP domain containing 1, and CLASP1 = the cytoplasmic linker-associated protein 1.

**Table 4:** Comparisons of the genomic prediction accuracy with standard error among genotyping platforms according to the Bayesian methods in each response variable.

Trait ^1^	Response Variables ^2^	Genotyping Platforms	π ^3^	BayesB	BayesC
IBW	DEBVexcPA	Axiom55K	0.99	0.188 (0.014)	0.178 (0.013)
		Illumina60Kv2	0.99	0.168 (0.012)	0.157 (0.012)
		Axiom660K	0.999	0.163 (0.013)	0.150 (0.013)
	DEBVincPA	Axiom55K	0.99	0.261 (0.012)	0.252 (0.013)
		Illumina60Kv2	0.99	0.224 (0.013)	0.210 (0.013)
		Axiom660K	0.999	0.223 (0.012)	0.215 (0.013)

^1^ IBW = individual birth weight; ^2^ DEBVexcPA = DEBV-excluding parent average and DEBVincPA = DEBV-including parent average; ^3^ π = distribution value.
